# Mitophagy Activation by Urolithin A to Target Muscle Aging

**DOI:** 10.1007/s00223-023-01145-5

**Published:** 2023-11-05

**Authors:** Julie Faitg, Davide D’Amico, Chris Rinsch, Anurag Singh

**Affiliations:** Amazentis SA, EPFL Innovation Park, 1024 Ecublens, Switzerland

**Keywords:** Urolithin A, Aging, Mitochondria, Mitophagy, Muscle health

## Abstract

The age-related loss of skeletal muscle function starts from midlife and if left unaddressed can lead to an impaired quality of life. A growing body of evidence indicates that mitochondrial dysfunction is causally involved with muscle aging. Muscles are tissues with high metabolic requirements, and contain rich mitochondria supply to support their continual energy needs. Cellular mitochondrial health is maintained by expansing of the mitochondrial pool though mitochondrial biogenesis, by preserving the natural mitochondrial dynamic process, via fusion and fission, and by ensuring the removal of damaged mitochondria through mitophagy. During aging, mitophagy levels decline and negatively impact skeletal muscle performance. Nutritional and pharmacological approaches have been proposed to manage the decline in muscle function due to impaired mitochondria bioenergetics. The natural postbiotic Urolithin A has been shown to promote mitophagy, mitochondrial function and improved muscle function across species in different experimental models and across multiple clinical studies. In this review, we explore the biology of Urolithin A and the clinical evidence of its impact on promoting healthy skeletal muscles during age-associated muscle decline.

## Introduction

Skeletal muscle aging is associated with progressive muscle mass and strength decline. Reducing muscle function below critical thresholds results in a clinical condition known as sarcopenia, significantly impacting quality of life by reducing the ability to perform daily tasks. Currently, the most efficient preventive and non-pharmacological strategies to attenuate sarcopenia are physical activity [1, 2] and caloric restriction (CR) [3–6]. Pharmacological interventions, such as myostatin inhibitors [7], and nutritional strategies, including high-protein or amino acid supplementation [8, 9], have focused on boosting muscle mass rather than muscle strength to circumvent the declining muscle function associated with aging. An effective strategy to improve muscle strength and function is via targeting muscle bioenergetics i.e., to enhance mitochondrial health via targeted interventions.

Mitochondrial health refers to the well-being and proper functioning of mitochondria, the organelles in most eukaryotic cells responsible for producing energy through adenosine triphosphate (ATP). Improving and maintaining mitochondrial health are considered potential strategies to enhance the health span, meaning how many of those years we remain healthy and disease-free. Mitochondrial health is crucial for cellular energy production, and it depends on several integrated processes that require multiple activities. These processes include Oxphos, protein import, and calcium uptake etc. The molecular components that perform these activities, such as enzymatic activities, protein transport and complexes, and Fe buffering, among others, defined in Monzel et al. [10]. Mitophagy serves as a quality control mechanism to maintain mitochondrial health by selectively removing dysfunctional or superfluous mitochondria from the cell. It ensures that only healthy mitochondria are preserved and can effectively contribute to cellular energy production and other essential functions.

Exercise and CR are two strategies to improve mitochondrial health [11]. However, both might suffer from poor compliance with exercise regimens and adherence to tedious caloric restriction protocols. We will summarize recent pre-clinical evidence and clinical studies that have shown the potential of the postbiotic (a gut microbiome-derived compound) Urolithin A (UA) to improve muscle function in different study populations such as overweight middle-aged and healthy older adults [12, 13] via its ability to activate mitophagy.

### Role of Mitophagy to Improve Mitochondrial Health in Sarcopenia

It is well established that mitochondria play a central role in skeletal muscle wasting during aging. The loss of strength and muscle mass in both old animals and older adults has been associated with impaired mitochondrial energy [14–16] and increased mitochondrial-mediated cell death by apoptosis [16–18]. One cause of the loss of mitochondrial homeostasis during aging is the accumulation of dysfunctional mitochondria. This is, in turn, derived from the impaired ability of cells to remove faulty organelles, a process called mitophagy [19]. As a result of damage to mitochondria or exposure to mitophagy inducers, mitochondria undergo mitophagy. Mitophagy is a selective autophagy process involving the clearing and recycling of dysfunctional mitochondria that are impaired with aging and in several age-related diseases.

The best-described regulatory pathway currently involves Parkin, an E3 ubiquitin ligase-like protein, in collaboration with PINK1. The PINK1-Parkin-dependent mitophagy process is initiated by stabilizing the kinase PINK1, which stimulates Parkin's recruitment and activation from the cytosol, unblocking its enzymatic activity and leading to the ubiquitination of outer membrane mitochondrial proteins [20]. The latter is recognized by an autophagic marker such as the microtubule-associated protein LC3 and recruit lysosomes containing hydrolytic enzymes, to form an autophagosome and degrade damaged mitochondria [21]. Other PINK1–Parkin-independent mitophagy pathways activate other mitochondrial proteins, such as BNIP3, FUNDC1, and NIX, directly recruit LC3 to promote autophagosome formation and mitochondrial degradation.

Mitophagy leads to improved mitochondrial respiratory capacity by improving the quality of cellular mitochondria pools. Extensive descriptions of mitophagy monitoring procedures, and their advantages and limitations, have been reviewed by Palikras et al., 2018 and Onishi et al., 2021 [22, 23].

### Urolithin A Intervention Mediated Mitophagy to Address Muscle Aging

UA is a member of the urolithin family, characterized by an alpha-benzo-coumarin scaffold in its chemical structure. It is a postbiotic metabolite derived from microflora conversion of ingested ellagitannins and ellagic acid, precursor molecules in foods like pomegranates, strawberries, raspberries, and walnuts. Having the right microbiome is required for Urolithin A’s natural conversion from dietary precursors. This occurs in approximately 30%–40% of people [24] at variable levels, with only a small percentage deriving UA from natural exposure at substantial levels able to confer a health benefit. This makes direct supplementation of UA a relevant strategy for everyone to benefit from this active molecule.

### Urolithin A Induced Mitophagy and Improves Muscle Function Pre-clinically

Data in cells, worms, mice, and humans showed that UA's most consistent effect is to improve mitochondrial health through mitophagy [12, 13, 25, 26]. In worms, UA enhanced the expression of mitophagy genes *lgg-1* [25], *pink-1*, and *pdr-1* [27] and exhibited a similar health span-promoting signature as CR. According to Ryu et al., the effect of UA is mediated also by skn-1 (Nrf2 homolog), which encodes for a transcription factor regulating mitochondrial biogenesis and mitophagy gene expression [25].

In wild-type rodents [25] and in the *mdx* mouse model of Duchenne muscular dystrophy (DMD) [27], higher levels of mitophagy markers such as mitochondrial Parkin, ubiquitinated and phospho-ubiquitinated mitochondrial proteins were observed in skeletal muscle tissues after UA supplementation. Also, complex I and II-mediated respiration in muscle tissues from *mdx* mice have been enhanced after UA supplementation [27]. Finally, when administered intragastrically to middle-aged mice for 16 weeks, UA increased markers of angiogenesis in the skeletal muscle, activated the SIRT1-PGC-1α pathway, and elevated ATP and NAD + levels[28]. UA-mediated mitophagy activation has been confirmed in several other tissues and experimental conditions [29–31].

**Fig. 1:**
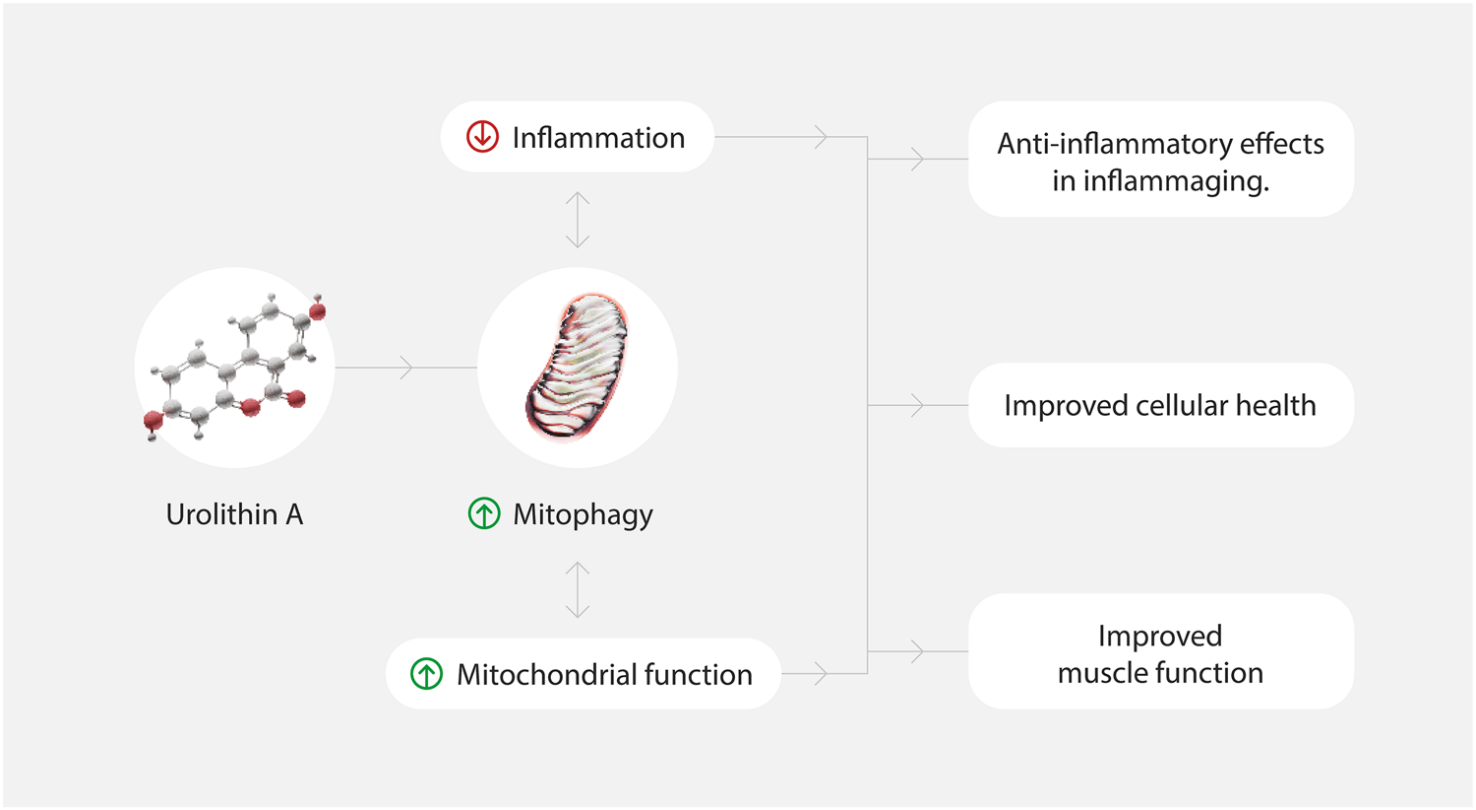
Beneficial effects of Urolithin A

Such improvements of mitochondrial health by UA supplementation have been associated with better health span in worms and rodent models (Fig. 1).

In *C. elegans*, UA led to an improvement of pharyngeal pumping measured by counting the contraction of the pharynx during 30 s and nematode motilities. In old mice UA administration promoted a 9% increase in muscle strength measured as grip test and a 57% increase of spontaneous activity (endurance) measured using the running wheel as compared with vehicle [25]. Notably, UA significantly increased aerobic endurance in young rats undergoing voluntary running activity, indicating that addition of UA to an exercise regimen further improves muscle health benefits in this pre-clinical model.

Enhancement in muscle strength, endurance and tetanic force were observed with UA also in the *mdx* dystrophic mice model and UA significantly increased the survival rate in the more severe DKO mice model of DMD [27].

Finally, UA-induced mitophagy and protected against cartilage degradation in a rat osteoarthritis (OA) disease model [32] and in human chondrocytes obtained from OA patients.

These pre-clinical data have shown that UA can activate mitophagy through either PINK1-Parkin dependent or independent mitophagy [33] and indicate a combined effect of UA on the skeletal-muscle system to protect against age-related decline in mobility, sarcopenia, and to support healthy mobility (Table 1). These findings have paved the way for investigating the role of UA in different human clinical settings.

### UA in Human Randomized Clinical Trials

In humans, UA was shown to regulate mitochondrial function systemically and in skeletal muscle.

The effect of oral administration of UA has been investigated in multiple double-blind, randomized, placebo-controlled clinical trials. The first-in-human trial (NCT02655393) enrolled healthy older adults, males and females, aged 61–85 years (mean age ~ 70 years). Subjects were administered either placebo, UA 500 mg, or UA 1000 mg either with single ascending doses or in multiple dosing for 4 weeks. First, this study investigated UA’s safety, tolerability and pharmacokinetic profile in older adults. UA exhibited a very safe profile, showed a dose-dependent linear increase in bioavailability up to the 1000 mg dose and did not accumulate in the plasma over time. Following intake, UA peaked in plasma between six and eight hours and exhibited a relatively long half-life (*t*1/2 = 17–22 h), probably because it was actively recirculated through the liver. There was also no food-effect on UA bioavailability.

In the multiple ascending dosing part of the study, different cohorts received either a placebo or two doses—500 and 1000 mg—of UA daily for four weeks. Muscle tissue collection and blood sampling for biological markers of mitochondrial function were performed at the baseline visit and after four weeks following the last UA dose. In the *vastus lateralis* skeletal muscle, UA enhanced the expression of several mitochondrial function-related gene sets in dose dependent manner [26]. Interestingly, the same gene sets were lower in the muscles of pre-frail older adults compared with healthy older adults who practiced regular physical activity [34]. Specific genes related to mitophagy activity, such as PARK2GABARAPL1and Ulk1were also positively impacted by UA in the muscle. In plasma, UA administration of up to 1000 mg daily led to a decrease in several plasma acylcarnitines, which are markers that reflect the efficacy of mitochondrial fatty acid oxidation [26]. The findings of this first clinical trial confirmed results observed in pre-clinical studies, demonstrating UA's safety, bioavailability, and positive effect on muscle mitochondria.

This first-in-human study was followed by two efficacy studies with UA supplemented for a longer duration (2–4 months) in which subjects were tested, for the first time for, a battery of muscle function and physical performance outcomes The first efficacy study investigated UA’s benefits on muscle endurance in healthy older adults (aged 65–90 years, mean age ~ 72 years) after daily administration of UA 1000 mg for 4 months (ClinicalTrials.gov Identifier: NCT03283462). Subjects were selected based on low mitochondrial function in their hand skeletal muscle (*first dorsal interosseus* muscle strength assessed via magnetic resonance spectroscopy) and low-average physical performance (assessed via the 6-min walk test) [13]. The primary outcome of the study was the change in six-minute walking distance from the baseline after 4-months of UA supplementation. A key secondary outcome was the change in muscle endurance (resistance to fatigue) via single muscle function tests in hand and leg muscles. Walking distance was greater in the UA group, but compared to the placebo group these changes were not-significant, suggesting that intervention longer than 4-months with a higher sample size would be needed in future studies. Interestingly clinical data showed UA to increase by ~ 20% the resistance to fatigue of both hand (*first dorsal interosseus*) and leg (*tibialis anterior*) skeletal muscles, after 2 months, compared to the placebo group, The positive effect of UA on resistance to fatigue was maintained after 4-months of supplementation. Given that these are two functional and anatomically different muscles, UA was likely to have a global impact on skeletal muscle mitochondria. Notably, the improvement in fatigue resistance with UA occurred without any changes to participants’ exercise routine and dietary habits.

At biomarker levels, these clinical effects were associated with a significant decrease in circulating plasma acylcarnitine levels, consistent with data observed from the first-in-human study. UA oral intake also reduced plasma biomarkers of inflammation, such as ceramides [35], and C-reactive protein [36] compared to placebo [13]. These biomarkers are known to be elevated with chronic inflammation observed during aging, a process known as “inflamm-aging” [37].

The improvement in fatigue resistance occurred without any changes to participants' exercise routine and dietary habits and indicated the efficacy of UA to counteract muscle aging decline in older adults. This study confirmed that UA was safe and bioavailable following long-term supplementation.

Another randomized, double-blind, placebo-controlled study tested the efficacy of UA in a middle-aged population (Singh et al., 2022). The recruited subjects (aged 40–64 years, mean age ~ 52 years) had low endurance (Vo2max < 25 ml/kg/min) and were overweight (~ 29 kg/m^2^ BMI). In this study, subjects were randomized to one of three interventional groups: Placebo, 500 mg of UA, and 1000 mg of UA. The duration of administration was 4 months. Plasma biomarkers of mitochondrial health, muscle strength measures, 6-min’ walks, and peak VO2 were evaluated. UA at both doses significantly improved leg muscle strength by ~ 10%–12%, compared to placebo. It also resulted in better aerobic capacity, measured as peak Vo2 (improved by 10%), and physical performance measured as a 6-min walk test (7% improvement in walking distance). As in the two previous studies, UA significantly decreased plasma acylcarnitine levels compared to baseline levels at 500 mg, indicating better mitochondrial health at the systemic level and decreased circulating pro-inflammatory cytokines and CRP. In this trial, muscle biopsies were also collected, allowing the investigation of molecular mechanisms associated with improved muscle function with UA. Unbiased proteomic analysis of the muscle biopsy samples revealed that UA’s most significantly enriched pathway at 500 mg was “Parkin-mediated ubiquitin and proteasomal systems” [12]. These findings indicated for the first time a UA-mediated molecular signature of increased mitophagy in the skeletal muscle in humans following long-term supplementation. These results were further validated by targeted protein analysis, with an increase by UA in the phosphorylation level of Parkin on Serine 65 [12], a marker of Parkin activation and mitophagy levels [38]. Proteomics and western blot analysis of the 1000 mg cohort showed that UA increased levels of oxidative phosphorylation enzymes, which are proteins linked to improved mitochondrial function. UA at this dose also increased mitochondrial abundance, measured by mitochondrial over DNA content (mtDNA/DNA). These data confirmed UA’s ability to promote human muscle and mitochondrial health.

Altogether, these clinical studies [12, 13, 26] consistently indicate that oral UA, in the range of 500–1000 mg, increases mitophagy and mitochondrial metabolism in skeletal muscle, and report positive effects on a range of muscle function outcomes, including skeletal muscle resistance to fatigue, improved muscle strength, and exercise performance measures (Fig. 1, Table 1).

**Table 1:** Summary of pre-clinical and clinicals studies on UA and muscle health

	Species	Age	Specific effects	References
Pre-clinical	*Caenorhabditis elegans*		↑Mitophagy ↑Mobility ↑Pharyngeal pumping rate ↑Lifespan	[25]
	Wild Type Mice	*23 months* *16 months*	↑Endurance ↑Grip strength ↑Mitophagy ↑Muscle function	[25]
	Rats	*5.5 weeks*	↑Running activity	
	*mdx* mice	*5 weeks*	↑Grip strength ↑Tetanic force ↑Running activity	[27]
Clinical	Humans	*61–85 years* *Healthy elderly*	↓Acylcarnitines ↑Mitochondrial gene expression	[26]
		*65–90 years* *Healthy elderly*	↑Resistance to fatigue ↓Acylcarnitines ↓CRP ↓Ceramides	[13]
		*40–64 years* *Healthy overweight*	↑Muscle strength ↑Muscle endurance ↓Acylcarnitines ↓CRP ↑ Mitophagy and mitochondrial respiration proteins	[12]

### Future Perspectives

Altogether, the data gathered herein support the health-promoting activity of UA. Several ongoing studies are further investigating the impact of mitophagy activation via UA in additional clinical settings.

An ongoing RCT will examine the impact of UA supplementation on muscle performance and recovery parameters in high-performance athletes undergoing a controlled exercise training program (NCT04783207). Another trial is investigating the impact of UA on the mitochondrial health of immune cells and overall immune function in a healthy, middle-aged population (NCT05735886) and yet another is investigating the impact of combining UA with high-protein supplementation in an immobilization setting that rapidly induces muscle atrophy (NCT05814705).

It would be interesting for future trials to investigate the association between ability to naturally produce UA from diet and muscle function with aging. Furthermore, human studies should be designed to study the impact of UA on other organs impacted by aging and that could contribute to disability during sarcopenia. For instance, joint and heart muscle tissues for which promising preclinical data on a beneficial impact of UA are already available. Curent studies and the growing research on UA indicate how supplementing with UA is an effective nutritional intervention to counter age-related muscle decline, by targeting mitophagy (Table 1).
